# Natural Oils as Green Solvents for Reactive Extraction of 7-Aminocephalosporanic Acid: A Sustainable Approach to Bioproduct Recovery in Environmental Biotechnology

**DOI:** 10.3390/biom15101371

**Published:** 2025-09-26

**Authors:** Delia Turcov, Madalina Paraschiv, Alexandra Cristina Blaga, Alexandra Tucaliuc, Dan Cascaval, Anca-Irina Galaction

**Affiliations:** 1 Department of Biomedical Sciences, Faculty of Medical Bioengineering, Grigore T. Popa University of Medicine and Pharmacy Iasi, M. Kogalniceanu 9-13, 700454 Iasi, Romania; delia.turcov@umfiasi.ro (D.T.); anca.galaction@umfiasi.ro (A.-I.G.); 2 Department of Organic, Biochemical and Food Engineering, Cristofor Simionescu Faculty of Chemical Engineering and Environmental Protection, Gheorghe Asachi Technical University of Iasi, D. Mangeron 73, 700050 Iasi, Romania; alexandra-cristina.blaga@academic.tuiasi.ro (A.C.B.); alexandra.tucaliuc@academic.tuiasi.ro (A.T.); dan.cascaval@academic.tuiasi.ro (D.C.)

**Keywords:** 7-aminocephalosporanic acid, reactive extraction, natural oils, green solvents, bioproduct recovery, sustainable bioprocessing

## Abstract

The growing need for environmentally friendly separation processes has motivated the search for alternative solvents to petroleum-derived chemicals for the recovery of biosynthesized products. Although effective, conventional petroleum-based solvents pose major environmental and sustainability concerns, including pollution, ecotoxicity, human health risks, and high costs and energy demands for recycling. Consequently, current research and industrial practice increasingly focus on their replacement with safer and more sustainable alternatives. This study investigates the use of natural oils (i.e., grapeseed, sweet almond, and flaxseed oils) as renewable, biodegradable, and non-toxic diluents in reactive extraction systems for the separation of 7-aminocephalosporanic acid (7-ACA). The combination of these oils with tri-n-octylamine (TOA) as extractant enabled high extraction efficiencies, exceeding 50%. The system comprising 120 g/L tri-n-octylamine in grapeseed oil, an aqueous phase pH of 4.5, a contact time of 1 min, and a temperature of 25 °C resulted in a 7-ACA extraction efficiency of 63.4%. Slope analysis suggests that complex formation likely involves approximately one molecule each of tri-n-octylamine and 7-ACA, although the apparent order of the amine is reduced in systems using natural oils. This study highlights the potential of natural oil-based reactive extraction as a scalable and environmentally friendly method for 7-ACA separation, aligning with the principles of green chemistry and environmental biotechnology.

## 1. Introduction

Cephalosporins are widely used as part of the β-lactam antibiotics class, produced by fungi *Acremonium chrysogenum* (formerly *Cephalosporin acremonium*), nowadays with 5 generations of drugs currently being developed. These compounds are derived from 7-aminocephalosporanic acid, which serves as a precursor for the second and third generation semisynthetic derivatives [1,2]. 7-ACA can be easily modified at the 7-position of the chain, and thus serves as a versatile building block for several important antibiotics like cephalexin, cefadroxil and ceftriaxone [2,3].

Traditionally, 7-ACA was produced by chemical deacylation of cephalosporin C (CPC), a process with harsh conditions and usage of dangerous reagents like phosphorus pentachloride or nitrosyl chloride, resulting in high waste generation and modest yields [2,4]. Over the past three decades, this conventional method has been predominantly replaced by enzymatic processes involving D-amino acid oxidase (DAAO), glutaryl acylase (GA), or co-immobilized enzyme systems [5,6]. The biocatalytic processes have conversion efficiencies greater than 90%, besides greatly reducing environmental impacts—lowering the waste from over 30 kg to less than 1 for each kg of 7-ACA obtained [1].

However, the downstream processing of 7-ACA from aqueous media from post-enzymatic reaction mixtures remains a challenging task. Its amphoteric character, high water solubility, and sensitivity to pH changes, hinder efficient isolation, especially in the presence of structurally similar byproducts like glutamic acid or α-aminoadipic acid [2,7]. Conventional purification methods, including isoelectric precipitation (around the pH value of 3.5), ion-exchange chromatography, and resin adsorption, often have poor selectivity and unsatisfactory scalability [1,8].

Within this framework, reactive extraction has shown great efficiency and technical feasibility in the separation of biosynthetically produced compounds, with major improvements compared to traditional downstream processing methods. Its primary advantage consists of the considerable enhancement of extraction yield by adding specific extractants in the organic phase, which allows the selective recovery of ionizable substances like carboxylic acids, amino acids, or antibiotic precursors directly from fermentation broths. Through this method, several purification steps are eliminated, energy is saved, and the generation of solid and liquid wastes is minimized, fitting the principles of “white biotechnology” and sustainable processing. In addition, reactive extraction can be customized by proper selection of extractants (e.g., amines, organophosphorus compounds) and diluents in order to maximize solute—extractant interactions and optimize phase separation. In the selective recovery of amphoteric and ionic compounds from dilute aqueous solutions, this method has proven highly effective. For 7-aminocephalosporanic acid, extraction mechanisms vary significantly depending on the type of extractant used. When quaternary ammonium extractants, such as Aliquat 336, are employed, the process relies on the reversible formation of ion pairs between the deprotonated (anionic) form of 7-ACA and the permanently charged cationic headgroup of the extractant. In such systems, the extraction efficiency is typically optimized in an alkaline pH range (7.5–8.5), where the carboxyl group of 7-ACA is fully deprotonated, enabling strong electrostatic interactions. Subsequent back-extraction into an acidic aqueous phase (pH < 4) facilitates the regeneration of the extractant and recovery of the target compound [2,7,9,10,11,12,13]. In contrast, secondary and tertiary amines are not permanently charged and require protonation in acidic media to form extractable complexes with 7-ACA. Borra et al. systematically studied the reactive extraction of 7-ACA using secondary, tertiary, and quaternary amines, reporting that extraction with secondary and tertiary amines is most effective in the mildly acidic to neutral pH range (pH 5–8), where 7-ACA remains relatively stable. In these systems, the distribution coefficient decreases with increasing pH, indicating that the extraction is favored under conditions where the amine is protonated [7].

Although promising, an important drawback of the method concerns the selection of organic solvents, which are frequently toxic, volatile, or environmentally persistent. While effective, these approaches raise critical concerns regarding environmental safety and long-term sustainability, as they are associated with excessive wastewater generation, air pollution, ground-level ozone formation, contribution to climate change, ecotoxicological effects, and adverse impacts on human health. Moreover, the recycling of such solvents entails considerable economic costs and high energy consumption. In response to these challenges, contemporary research and industrial practice increasingly emphasize the substitution of petroleum-derived solvents with environmentally benign and sustainable alternatives [14,15]. Common organic solvents such as dichloromethane, hexane, heptane, and ethyl acetate are widely employed in extraction systems owing to their favorable physicochemical properties, which provide high solubility and extraction yields. However, their use raises significant concerns due to potential toxicities and associated health risks. For instance, dichloromethane, previously categorized as Group 2B “possibly carcinogenic to humans”, has recently been reclassified by the International Agency for Research on Cancer (IARC) to Group 2A, indicating it is “probably carcinogenic to humans” [14,16,17]. In this context, the central challenge of green extraction lies in replacing conventional organic solvents with sustainable alternatives, including renewable natural products, while simultaneously reducing energy consumption and unit operations to ensure safe, efficient, and high-quality products [18,19]. The application of natural oils as bio-based solvents for the extraction of biosynthesized compounds presents a significant edge over traditional petroleum-based solvents, conforming to the principles of green chemistry and sustainable processing. Sunflower, rice bran, soybean, mustard, and palm oils are renewable, biodegradable, and generally recognized as safe (GRAS), making them highly suitable for food, pharmaceutical, and biotechnological applications where low solvent toxicity is essential [20,21].

These oils function as non-volatile, hydrophobic diluents and can be combined with extractants (e.g., tri-n-butyl phosphate, TBP) to enable the selective recovery of ionizable compounds, such as organic acids, amino acids, and antibiotic intermediates, directly from fermentation broths. The use of TBP in sunflower oil achieved high extraction efficiency for vanillic acid, with guarantees of process safety and environmental risk aversion. Similarly, natural oils have been employed for itaconic acid extraction from aqueous solutions (91.52% extraction efficiency was achieved by using a mixture of tri-n-octylamine with rice bran oil), showing a high level of selectivity and low co-extraction of byproducts, compared to petrochemical solvents [21,22]. In bioprocessing and fermentation applications, the use of bio-based oils as diluents is considered a less toxic and more biocompatible alternative for in situ separations, allowing direct extraction from fermentation broths without adversely affecting microbial viability. This advantage has been specifically demonstrated in the reactive extraction of organic acids using natural oils such as sunflower oil, rice bran oil, and mustard oil [21]. Extraction systems based on mixtures of natural oils and tri-n-octylamine have gained increasing attention and have been successfully applied for the recovery of several other carboxylic acids, including pyruvic acid, lactic acid, propionic acid, butyric acid, and gallic acid. Lactic acid was efficiently recovered from aqueous solutions with a maximum separation efficiency of 74.54% using rice bran oil as the solvent in combination with TOA at a concentration of 3.61 mol·kg^−1^. Similarly, soybean oil, another vegetable oil recognized for its non-toxicity and microorganism-friendly properties, was used in the reactive extraction of propionic acid from fermentation broth, yielding a distribution coefficient of 0.73 and an extraction efficiency of 42.25%. In the case of gallic acid, reactive extraction from aqueous solutions using TBP and TOA as extractants dissolved in three non-toxic natural oils was investigated. The results demonstrated the extractability of solvents in the order sunflower oil  >  rice bran oil  >  soybean oil for TOA, and rice bran oil  >  sunflower oil  >  soybean oil for TBP [23,24,25,26,27,28].

In the food sector, vegetable oils have been employed as green solvents or co-solvents for the extraction of carotenoids and phenolic compounds from plant matrices. For example, corn oil has been successfully used in ultrasound- and microwave-assisted extraction of carotenoids, demonstrating the feasibility of edible oil-based systems as alternatives to toxic organic solvents [29].

Apart from selectivity and process compatibility considerations, natural oils possess several sustainability benefits, as they are biodegradable, non-toxic, and have no emission of volatile organic compounds. These attributes make natural oils safer and more environmentally acceptable than traditional solvents like hexane, dichloromethane, or toluene, which all pose significant risks of air and water pollution, human toxicity, and environmental persistence [20,23].

The current study proposes vegetable oils (sweet almond oil, flaxseed oil, and grapeseed oil) as innocuous, natural diluents for 7-ACA reactive extraction and compares their systematic performance against a traditional benchmark (dichloromethane), while investigating the effects of tri-n-octylamine as extractant. This study investigates whether bio-derived diluents, specifically vegetable oils, can achieve comparable efficiency and loading capacity, while offering enhanced sustainability and safety in downstream processes.

## 2. Materials and Methods

To analyze the separation of 7-ACA, the following chemicals were employed:

- 7-aminocephalosporanic acid (Sigma-Aldrich, Saint Louis, MO, USA, >98%), with initial concentration in the aqueous phase of 0.5 g/L (1.83 × 10^−4^ M);

- tri-n-octylamine as extractant (molecular weight, 353.67 g/mol; density, 0.81 g/cm^3^; batch number S37081 406) (Sigma-Aldrich, Saint Louis, MO, USA, >99%), with concentrations in the organic phase varying between 10 and 120 g/L (0.028–0.339 M);

- dichloromethane (Fluka, >99.9%, Seelze, Germany) (dielectric constant 9.08 at 25 °C) [30], sweet almond oil (batch number 9242, Germany), grapeseed oil (batch number 19242, Hungary), and flaxseed oil (batch number 20243, Belgium) (physical properties are presented in Table 1) (procured from Herbal Sana, Oradea, Romania) as bio-solvents. All oils used in this study were 100% pure, without any additives, with 0–0.10% water content, and were obtained by cold pressing followed by filtration. The oils were not refined or deodorized prior to use.

The pH values of the aqueous phase varied in the range of 2–9, maintained by using standard buffer solutions (citrate-phosphate buffer for pH 2–4, phosphate buffer for pH 6–8, and carbonate-bicarbonate buffer for pH 9) [7]. The pH values were determined using the digital pH meter (Consort C836 type, Consort, Turnhout, Belgium) and recorded throughout each experiment, with any change of pH value being monitored during the extraction experiments.

The experiments on the separation of 7-aminocephalosporanic acid by reactive extraction with tri-n-octylamine were carried out in a vibratory mixing extraction column, designed to ensure a large interfacial area and rapid attainment of steady-state conditions. The laboratory setup [32] consisted of a glass column (46 mm internal diameter, 250 mm height) equipped with a thermostatic jacket, through which a water–ethylene glycol mixture was circulated to maintain the temperature at 25 °C. Mixing of the aqueous and organic phases, in a volumetric ratio of 1:1 (20 mL of each phase), was achieved by means of a perforated disk (45 mm diameter, 20% free cross-sectional area) subjected to vertical vibrations at a frequency of 50 s^−1^ and amplitude of 5 mm, positioned at the initial interface between the aqueous and organic phases. Each extraction experiment was conducted for 1 min, after which the resulting emulsion was separated in a Nahita centrifugal separator operating at 10,000 rpm.

The concentration of 7-ACA in the aqueous phase was determined by HPLC analysis (Dionex Ultimate 3000, Thermo Fisher Scientific Inc., Waltham, MA, USA), on a C8-Aquapore RP-300 column (7 µm, 250 × 4.6 mm). The mobile phase consisted of 25 mM KH_2_PO_4_, pH 3.0, and 5% acetonitrile as elution buffer. The flow rate was set to 1 mL/min, the column temperature maintained at 25 °C, and the injection volume was 10 μL. The analyte was detected at 254 nm (Calibration curve presented as Figure S1 Supplementary Materials) [33].

To validate the reliability of the results, the experiments were conducted in triplicate. Statistical analysis indicated that the average values were consistent, with deviations not exceeding 5.48%, thereby confirming the reproducibility of the data.

## 3. Results

**Figure 1:**
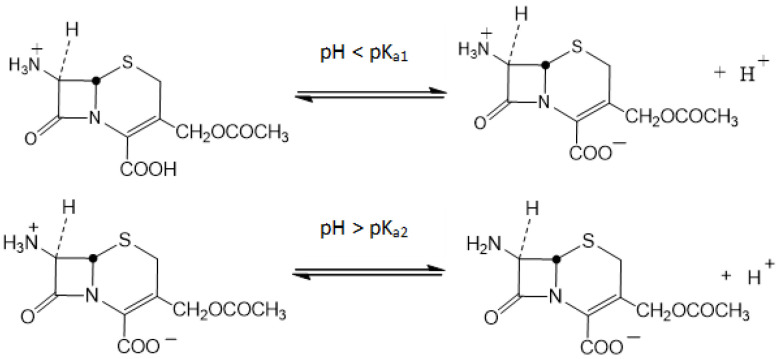
Ionic forms of 7-ACA as a function of pH.

According to the scientific literature, 7-ACA exists in different forms in aqueous solution depending on the pH of the medium (Figure 1). At pH values below 2.02 (pK_a1_ = 2.02), the molecule is predominantly cationic, above 4.42 (pK_a2_ = 4.42), the anionic form prevails, while within the intermediate range (2.02–4.42), the zwitterionic species is dominant [2,9].

In the aqueous phase, 7-ACA (*HA*) dissociates above its first pK_a_ via ionization of the carboxyl group to yield a proton (*H^+^*) and its corresponding anion (*A^−^*):

(1) $${HA}_{\left(aq\right)}↔\;H_{\left(aq\right)}^{+}+\;A_{\left(aq\right)}^{-}$$

The dissociation constant, $K_{a}$, is given by the following equation:

(2) $$K_{a}=\;\frac{\left[H^{+}\right]\;\left[A^{-}\right]}{\left[HA\right]}$$

where $\left[H^{+}\right]$, $\left[A^{-}\right]$, and $\left[HA\right]$ are the concentrations of $H^{+}$, $A^{-}$, and HA.

The reactive extraction occurs by means of the interfacial interactions between the solute and the extractant (${R_{3}N}_{\left(org\right)}$). The protonated amine ($R_{3}NH_{\left(org\right)}^{+}$) in the organic phase can form an extractable ion-pair with the anionic form of 7-ACA [34]:

(3) $${R_{3}N}_{\left(org\right)}+\;A_{\left(aq\right)}^{-}+\;H_{\left(aq\right)}^{+}\;↔\;{\left[{R_{3}NH}^{+}\;\cdot \;A^{-}\right]}_{\left(org\right)}$$

Depending on the molecular structures of the system components and the polarity of the solvent, either acidic or aminic adducts may form at the aqueous—organic interface. In line with observations from the reactive extraction of other carboxylic acids, the steric hindrance of the bulky 7-ACA acid molecule, combined with its low initial concentration compared with that of TOA, reduces the possibility of acidic adduct formation. Consequently, the interfacial complex is more likely to be of the ammonium salt type, arising from the neutralization of the solute carboxyl group with a single extractant molecule, or of the aminic adduct type involving multiple extractant molecules (*n* ≥ 2). Such molecular associations are favored in solvents of low dielectric constant, where they enhance the hydrophobic character of the interfacial complex [35,36].

(4) $$n\;{R_{3}N}_{\left(org\right)}+A_{\left(aq\right)}^{-}+n\;H_{\left(aq\right)}^{+}\;↔\;{\left[{\left(R_{3}NH^{+}\right)}_{n}\;\cdot \;A^{-}\right]}_{\left(org\right)}$$

A preliminary investigation was carried out to determine the optimal pH value for maximum extraction efficiency of 7-ACA with TOA by analyzing the influence of pH value of the aqueous phase on the separation efficiency of 7-ACA, using dichloromethane as the solvent, a medium widely employed in reactive extraction studies of carboxylic acids [13,32,37].

**Figure 2:**
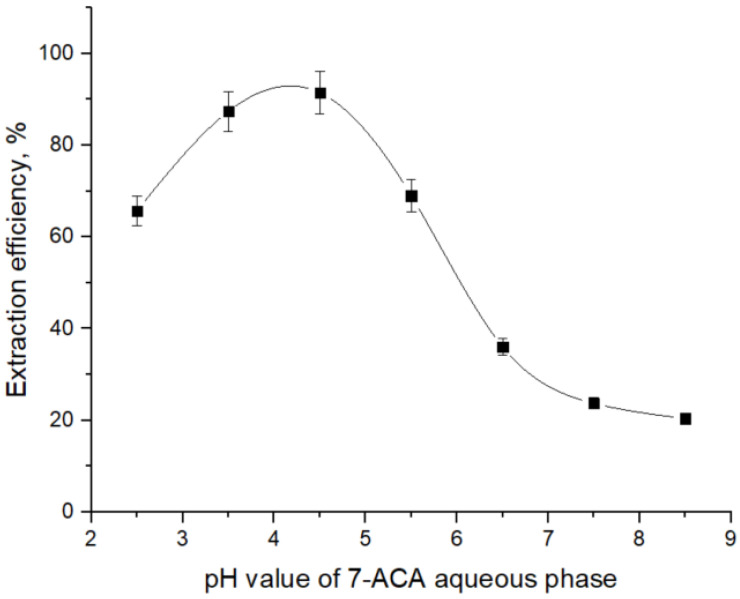
Effect of aqueous-phase pH on extraction efficiency of 7-ACA with 40 g/L TOA in dichloromethane.

From Figure 2 it can be seen that the extraction behavior of 7-ACA with tri-n-octylamine is strongly governed by the acid–base speciation of 7-ACA in the aqueous phase and the protonation state of TOA in the organic phase. As stated above, at pH values below pKₐ_1_ (= 2.02), 7-ACA exists predominantly in its cationic form, and therefore cannot effectively interact with protonated TOA ($R_{3}NH^{+}$), leading to low extraction yields. As the pH value increases into the interval between pK_a1_ and pK_a2_ (2.02–4.42), the zwitterionic form of 7-ACA becomes increasingly significant. In this region, the carboxylate anion of 7-ACA readily forms ion-pair complexes with $R_{3}NH^{+}$ in the organic phase, and thus the extraction efficiency increases. The extraction efficiency reaches a maximum at pH values of 4.42, where the concentration of the carboxylate anion is sufficiently high and the amino group is still predominantly protonated, stabilizing the ion-pair complex. At pH values above 4.42, deprotonation of the amino group reduces the zwitterionic stabilization of the molecule and its overall hydrophobicity decreases, resulting in higher aqueous solubility and reduced complexation efficiency. Consequently, the extraction yield declines at alkaline pH values.

To clarify the mechanism of 7-ACA acid extraction, it was assumed that the interfacial complex is formed by the interaction of one solute molecule with *n* molecules of the extractant, as previously described. Under this assumption, the extraction constant ($K_{E}$) can be calculated with the following equation:

(5) $$K_{E,n}=\;\frac{{\left[{\left(R_{3}{NH}^{+}\right)}_{n}\cdot A^{-}\right]}_{\left(org\right)}}{{\left[A^{-}\right]}_{\left(aq\right)}{\left[H^{+}\right]}_{\left(aq\right)}^{n}{\left[R_{3}N\right]}_{\left(org\right)}^{n}}$$

${\left[{\left(R_{3}{NH}^{+}\right)}_{n}\cdot A^{-}\right]}_{\left(org\right)}$—concentration of the 7-ACA-amine ion-pair complex in the organic phase;

${\left[A^{-}\right]}_{\left(aq\right)}$—concentration of the 7-ACA carboxylate anion in the aqueous phase;

${\left[H^{+}\right]}_{\left(aq\right)}$—proton concentration in the aqueous phase;

${\left[R_{3}N\right]}_{\left(org\right)}$—concentration of free tri-n-octylamine in the organic phase.

By assuming negligible physical extraction of undissociated 7-ACA, the distribution coefficient ($K_{D}$) for the reactive extraction of 7-ACA acid with TOA can be calculated by the expression:

(6) $$K_{D}=\;\frac{{\left[{\left(R_{3}{NH}^{+}\right)}_{n}\cdot A^{-}\right]}_{\left(org\right)}}{{\left[H_{2}A^{+}\right]}_{\left(aq\right)}+\;{\left[HA\right]}_{\left(aq\right)}+\;{\left[A^{-}\right]}_{\left(aq\right)}}$$

By combining Equations (5) and (6), the distribution coefficient becomes:

(7) $$K_{D}=\;K_{E,n}{\left[R_{3}N\right]}_{\left(org\right)}^{n}{\left[H^{+}\right]}_{\left(aq\right)}^{n}\frac{{\left[A^{-}\right]}_{\left(aq\right)}}{{\left[H_{2}A^{+}\right]}_{\left(aq\right)}+\;{\left[HA\right]}_{\left(aq\right)}+\;{\left[A^{-}\right]}_{\left(aq\right)}}$$

The distribution coefficient referred to the total aqueous concentration of 7-ACA, $K_{D},$ is defined as the ratio between the concentration of the ion-pair complex extracted into the organic phase, ${\left[{\left(R_{3}{NH}^{+}\right)}_{n}\cdot A^{-}\right]}_{\left(org\right)}$, and the total concentration of 7-ACA remaining in the aqueous phase, ${\left[H_{2}A^{+}\right]}_{\left(aq\right)}+\;{\left[HA\right]}_{\left(aq\right)}+\;{\left[A^{-}\right]}_{\left(aq\right)}$. In this expression, ${\left[H_{2}A^{+}\right]}_{\left(aq\right)}$ represents the cationic form of 7-ACA, predominant at strongly acidic pH values (pH < pK_a1_ = 2.02), while ${\left[HA\right]}_{\left(aq\right)}$ corresponds to the zwitterionic form dominant between the two pK_a_ values. The anionic form, ${\left[A^{-}\right]}_{\left(aq\right)}$, prevails at pH values above pK_a2_ = 4.42 and is the reactive species responsible for forming the ion-pair complex with protonated TOA. The equilibrium is governed by the extraction constant $K_{E,n}$, which quantifies the affinity of *n* tri-n-octylamine molecules for one 7-ACA anion. Accordingly, the terms ${\left[R_{3}N\right]}_{\left(org\right)}^{n}$ and ${\left[H^{+}\right]}_{\left(aq\right)}^{n}$ denote the concentrations of free TOA in the organic phase and protons in the aqueous phase, respectively, each raised to the stoichiometric order *n*, since protonation of TOA is required to form $R_{3}{NH}^{+}$.

Starting from the equation

(8) $$\alpha_{A^{-}}=\frac{K_{a1}K_{a2}}{{\left[H^{+}\right]}^{2}+K_{a1}\left[H^{+}\right]+K_{a1}K_{a2}\;}$$

that present the anionic fraction for compounds with 2 pK_a_ values [38], and taking into account the dissociation equilibrium, the term $\alpha_{A^{-}}$ becomes: $\frac{{\left[A^{-}\right]}_{\left(aq\right)}}{{\left[H_{2}A^{+}\right]}_{\left(aq\right)}+\;{\left[HA\right]}_{\left(aq\right)}+\;{\left[A^{-}\right]}_{\left(aq\right)}}$, accounting for the proportion of 7-ACA present in the reactive carboxylate form at a given pH value.

The distribution model can be rearranged to highlight the dependence of the extraction efficiency on the concentration of TOA. By introducing the anionic fraction $\alpha_{A^{-}}$ in Equation (7), the distribution coefficient becomes:

(9) $$K_{D}=\;K_{E,n}\;{\left[R_{3}N\right]}_{\left(org\right)}^{n}{\left[H^{+}\right]}_{\left(aq\right)}^{n}\alpha_{A^{-}}$$

In practice, $K_{D}$ was determined from the experimental concentrations in the aqueous and organic phases at equilibrium. For equal phase volumes (1:1), the extraction efficiency was calculated as $E=100\cdot K_{D}/\left(1+K_{D}\right)$, consistent with the reported values. To allow reproducibility of the speciation-based model, the anionic fraction $\alpha_{A^{-}}$ was explicitly calculated using Equation (8), yielding a value of 0.545 at pH 4.5. This value was then used in the calculation of $ln\left(\frac{K_{D}}{\alpha_{A^{-}}}\right)$, ensuring consistency between experimental data and the theoretical framework.

The correlation (9) represents in logarithmic form the equation of a straight line:

(10) $$ln\left(\frac{K_{D}}{\alpha_{A^{-}}}\right)=(lnK_{E,n}+n\;ln{\left[H^{+}\right]}_{\left(aq\right)})+n\;ln{\left[R_{3}N\right]}_{\left(org\right)}$$

The relation (10) allows plotting $ln\left(\frac{K_{D}}{\alpha_{A^{-}}}\right)\;$versus $ln{\left[R_{3}N\right]}_{\left(org\right)}$, where the slope corresponds to the apparent amine order *n*, and the intercept contains information on both the extraction constant and the proton concentration.

**Figure 3:**
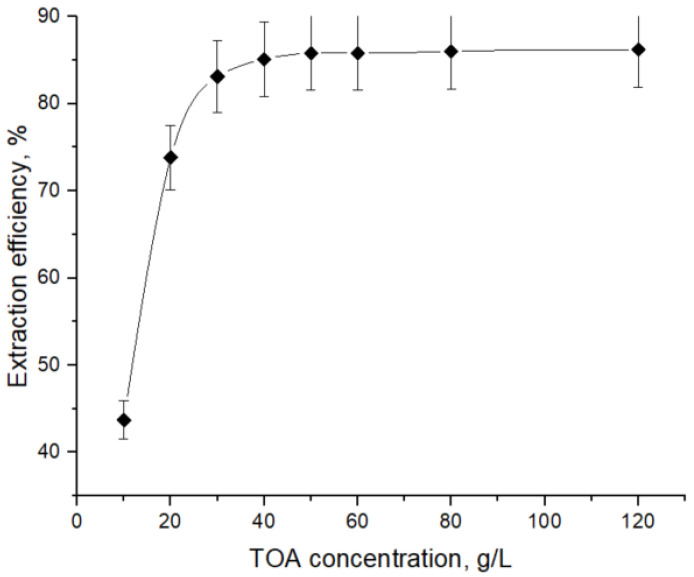
Effect of TOA concentration on reactive extraction efficiency of 7-ACA with dichloromethane.

To determine the number of aminic extractant molecules involved in the complexation with 7-ACA, the dependence of extraction efficiency on TOA concentration was investigated in dichloromethane, and the corresponding experimental trend is presented in Figure 3. The pH value of the aqueous phase was monitored and kept constant throughout the experiments, the chosen value being 4.5, where 7-ACA anion fraction is significant. A notably high extraction efficiency was obtained at pH 4.5, suggesting this value may be close to the optimum under the studied conditions.

**Figure 4:**
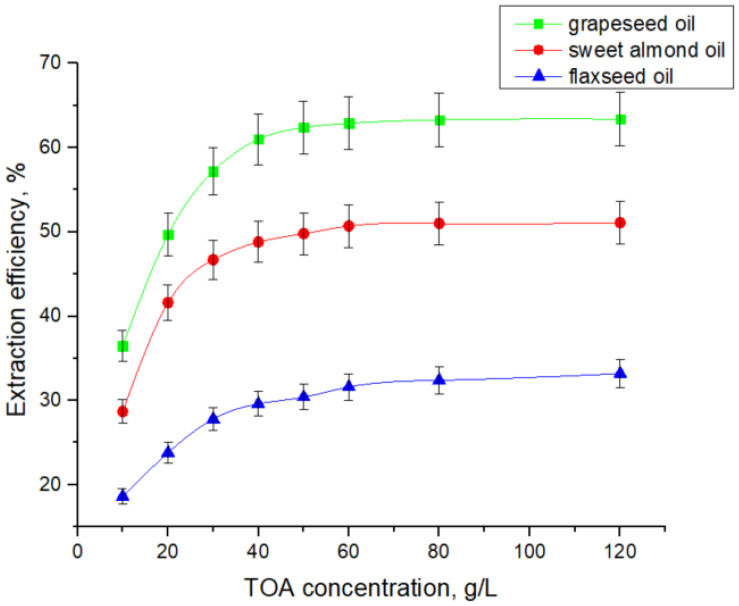
Effect of TOA concentration on reactive extraction efficiency of 7-ACA with natural oils.

In order to replace conventional toxic solvents such as dichloromethane, natural oils have been investigated as environmentally benign diluents in the reactive extraction system, offering advantages such as biocompatibility and sustainability. Figure 4 presents the influence of extractant concentration on the extraction efficiency of 7-ACA with TOA dissolved in grapeseed, sweet almond, and flaxseed oils.

The results show that an increase in extractant concentration in the solvent phase has a positive effect on acid extraction, as it enhances the interfacial availability of one of the reactants. As shown in Figure 3 and Figure 4, the influence of TOA concentration is significant only up to a threshold value, beyond which further increases exert little effect. The critical amine concentration at which this transition occurs depends on solvent polarity, being around 30–40 g/L for dichloromethane, but shifting to approximately 80 g/L in natural oils. This variation can be attributed to the polarity of the solvents, as lower polarity generally hinders solvation of charged ion-pairs. Also, the higher viscosity of natural oils used as solvents slows diffusion and interfacial renewal [21,22,39]. The extraction yields registered for extraction systems TOA—natural oils (63.4% for grapeseed oil, 51% for sweet almond oil, and 32% for flaxseed oil) were lower than the maximum yield obtained by using dichloromethane (86.2%).

**Figure 5:**
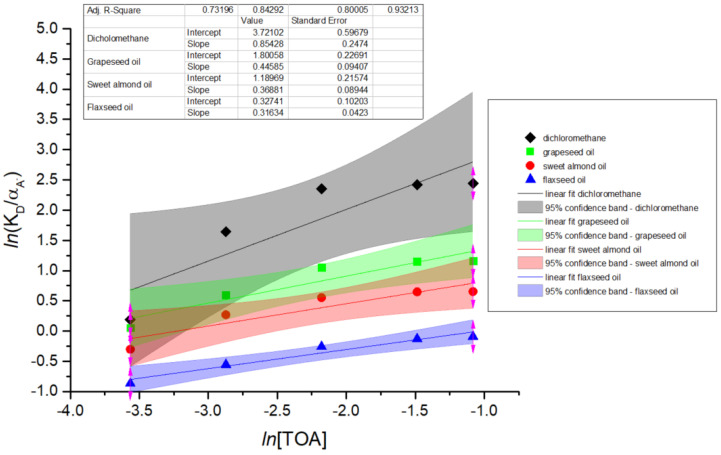
Graphical representation of the straight lines given by Equation (10) for dichloromethane, grapeseed oil, sweet almond oil, and flaxseed oil (pH = 4.5).

The data from Figure 3 and Figure 4, presented in detail in Table 2, Table 3, Table 4 and Table 5, were used for plotting in Figure 5 the straight lines described by Equation (10), for each solvent. The linear regressions were performed using all five TOA concentrations (expressed in mol/L) presented in Table 2, Table 3, Table 4 and Table 5, ensuring consistency between the experimental dataset and the calculated distribution parameters.

The values of the slopes calculated for all solvents used in the study correspond to the apparent stoichiometric order of TOA in the extracted complex. In the case of dichloromethane, *n* ≈ 1 indicates that the extraction proceeds predominantly via a 1:1 ion-pair complex between one 7-ACA carboxylate anion and one protonated TOA molecule, consistent with the mechanism proposed in the scientific literature [7]. The lower slope values observed in the presence of natural oils may indicate that solvation and/or mass-transfer limitations influence the extraction behavior, rather than reflecting a direct change in the effective amine order. The higher viscosity and lower polarity of these bio-based solvents are likely to reduce diffusivity and slow interfacial mass transfer, which can impact the apparent equilibrium behavior. Despite these limitations, natural oils remain highly attractive alternatives in reactive extraction processes, offering safer, biodegradable, and environmentally sustainable options that are consistent with green chemistry principles, while still maintaining competitive extraction performance.

**Figure 6:**
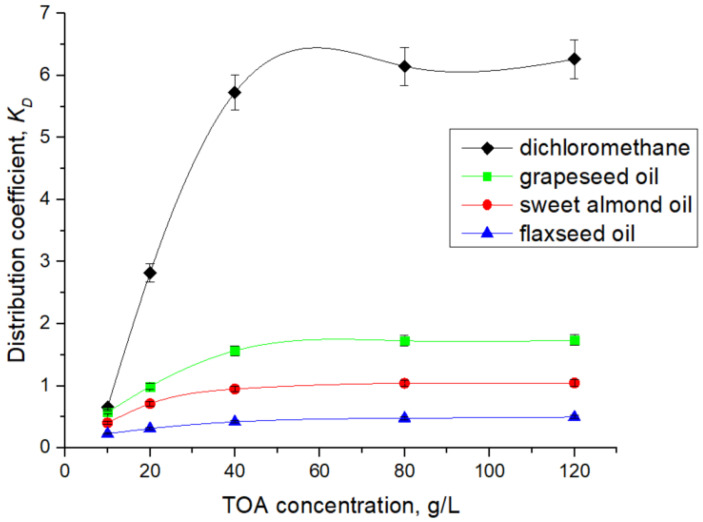
Effect of TOA concentration on the distribution coefficient, $K_{D}$.

As mentioned in Table 2, Table 3, Table 4 and Table 5 and represented in Figure 6, TOA in the range of 10 to 120 g/L (0.02–0.33 M) dissolved in dichloromethane enhanced the distribution coefficient, $K_{D}$, in the range of 0.77 to 6.26. Similarly, in the case of the natural oil solvents, the values of $K_{D}$ increase from 0.57 to 1.73 for grapeseed oil, from 0.40 to 1.04 for sweet almond oil, and from 0.22 to 0.49 for flaxseed oil. The variation in the distribution coefficient with TOA concentration reflects both the intrinsic affinity of the acid–amine interaction and the solvation capacity of the solvent. In dichloromethane, a polar aprotic diluent, $K_{D}$ increases steeply with TOA concentration, approaching saturation at relatively low concentrations, consistent with the predominance of a 1:1 ion-pair mechanism. In contrast, when natural oils are employed as diluents, the rise of $K_{D}$ with TOA concentration is markedly less pronounced. Grapeseed oil supports a moderate increase in $K_{D}$, while sweet almond oil and flaxseed oil display flatter trends, where higher TOA concentrations are required to achieve measurable gains in extraction efficiency.

The loading of the extractant, Z, is defined as the ratio between the total concentration of 7-ACA (summed over all molecular forms) present in the organic phase and the total concentration of the extractant (in all forms) in the organic phase at equilibrium. This parameter provides a direct measure of the utilization efficiency of the extractant: higher Z values indicate that a larger fraction of the extractant is actively complexed with the solute, while lower values reflect underutilization of the extractant or limitations imposed by solvent polarity, viscosity, or competitive equilibria at the interface [7]. The values of the loading factor, Z, registered for all the solvents used for the separation of 7-ACA, with variation in TOA concentration between 10 g/L and 120 g/L, was calculated according to Equation (11):

(11) $$Z=\;\frac{{\left[7-ACA\right]}_{\left(org\right)}^{tot}}{{\left[R_{3}N\right]}_{\left(org\right)}^{tot}}$$

${\left[7-ACA\right]}_{\left(org\right)}^{tot}$—the total concentration of 7-ACA in the organic phase at equilibrium;

${\left[R_{3}N\right]}_{\left(org\right)}^{tot}$—the total concentration of the extractant in the organic phase.

**Figure 7:**
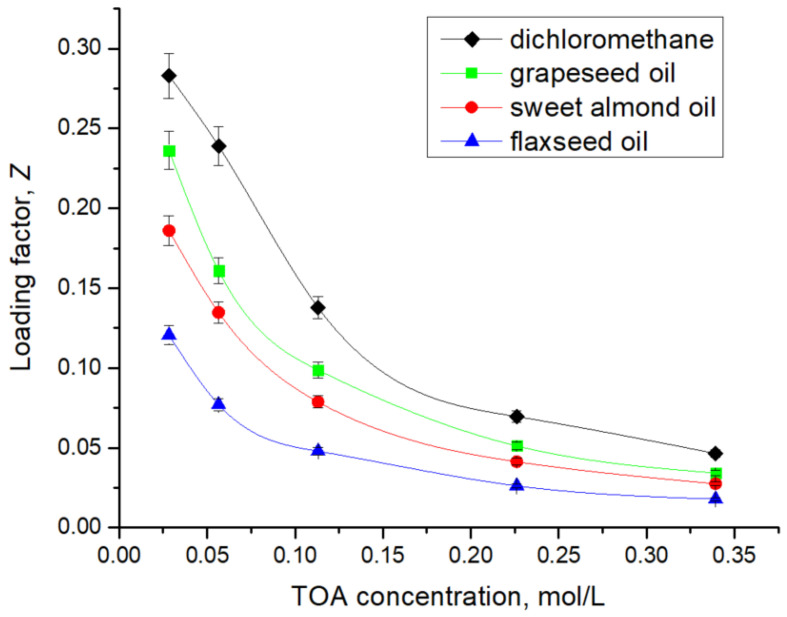
Effect of TOA concentration on the loading factor, Z.

The variation in the loading factor with TOA concentration, as illustrated in Figure 7, reveals clear differences among the solvents tested. In dichloromethane, Z reaches the highest values, confirming efficient utilization of TOA due to the favorable solvation of ion-pair complexes in a polar aprotic medium. In contrast, when natural oils are used as diluents, the values of Z decrease markedly with increasing TOA concentration. Grapeseed oil maintains higher loading efficiencies (0.236–0.034) compared to sweet almond oil (0.186–0.027) and flaxseed oil (0.120–0.017), which could be attributed to its relatively low viscosity and more favorable polarity for stabilizing the extracted complex. Sweet almond oil exhibits lower Z values, reflecting the role of higher viscosity in limiting interfacial mass transfer and reducing effective extractant participation. Flaxseed oil shows the lowest values across the concentration range, consistent with its very low polarity (only 1.4% polar lipids, glycol, and phospholipids [40]), which hinder ion-pair solvation and thus decrease extractant utilization efficiency. Overall, while dichloromethane enables the most efficient extractant loading, vegetable oils remain attractive alternatives as green solvents, balancing lower extraction efficiency with sustainability and safety.

Beyond extraction performance, the economic implications of solvent selection must also be considered when assessing process feasibility. From an economic perspective, the higher viscosity of bio-based solvents compared to petroleum-derived alternatives may result in elevated energy requirements for mixing and pumping operations, thereby increasing overall process costs. This trade-off highlights the importance of evaluating not only the technical efficiency of alternative solvents but also their impact on process sustainability and economic viability. While the viscosity of the natural oils used in this study vary between 30 and 58 cP at 25 °C (Table 1), conventional solvents such as kerosene or chloroform exhibit much lower viscosities (0.54–3 cP), which facilitate low-energy handling [41]. Each extraction experiment was carried out for only one minute, after which the phases were efficiently separated using a Nahita centrifugal separator at 10,000 rpm. The rapid phase disengagement observed under these short residence times indicates that the higher viscosities of the oils did not impose additional energy demands beyond standard centrifugal separation. Among the tested oils, flaxseed oil, with the lowest viscosity (30 cP), provided the most favorable balance between green solvent properties and process efficiency. Considering the financial implications for process design, food-grade oils are commercially available at prices typically ranging from €0.5 to €8 per kilogram, depending on quality and region [23,42,43]. These costs are comparable to those of several conventional organic solvents, while their non-toxicity and biodegradability confer further economic advantages by reducing expenses associated with handling and waste disposal, in addition to offering regulatory and safety benefits, particularly for pharmaceutical or food-related applications. Moreover, the use of renewable vegetable oils is consistent with the principles of green chemistry and contributes to the development of eco-compatible bioprocesses, a priority increasingly emphasized in both industrial practice and academic research.

## 4. Conclusions

The present study systematically evaluated the reactive extraction of 7-aminocephalosporanic acid (7-ACA) with tri-n-octylamine (TOA) using both a conventional solvent (dichloromethane) and three bio-based solvents (grapeseed oil, sweet almond oil, and flaxseed oil). The analysis included the influence of aqueous-phase pH value on extraction efficiency, the effect of TOA concentration on separation efficiency, as well as the determination of key extraction parameters such as the extraction constant $\left(K_{E}\right)$, distribution coefficient $\left(K_{D}\right)$, and loading factor (Z). The findings confirm that solvent polarity and viscosity exert a decisive role in controlling the efficiency of reactive extraction systems.

Dichloromethane exhibited the highest extraction efficiency and distribution coefficients, with slope values close to unity, consistent with the formation of a predominant 1:1 ion-pair complex between protonated TOA and deprotonated 7-ACA. The high intercept values further indicated strong affinity and efficient stabilization of the extracted complexes in this medium, yielding a maximum extraction efficiency of 86.2%. Although the yields obtained with vegetable oils were lower, both grapeseed oil and sweet almond oil achieved values above 50% (63.4% for grapeseed oil and 51.1% for sweet almond oil), confirming that, despite their higher viscosity and lower polarity compared to dichloromethane, these bio-based solvents can still provide practically relevant separation efficiencies. Flaxseed oil, in contrast, showed the lowest efficiency, reflecting its unfavorable physicochemical properties for stabilizing the extracted complexes.

Despite their comparatively lower efficiency, natural oils present significant advantages as alternative solvents. Their low volatility, biodegradability, and compatibility with biological systems make them environmentally safer than chlorinated solvents such as dichloromethane, which pose serious concerns of toxicity and persistence. Their use also aligns with the principles of green chemistry, reducing risks of solvent losses and enabling potential integration of extraction directly into bioprocesses without compromising microbial viability. These benefits highlight the potential of vegetable oils as sustainable carriers in the development of eco-friendly downstream processes.

Overall, this research demonstrates that while conventional solvents achieve higher extraction performance, bio-based oils present a promising balance between effectiveness and eco-friendliness. By combining moderate extraction yields with clear environmental and safety benefits, vegetable oils emerge as viable alternatives for the reactive extraction of 7-ACA and related carboxylic acid derivatives. Future research will aim to optimize oil–amine systems by adjusting operating conditions and exploring synergistic extractant combinations, thereby improving performance while maintaining the sustainability advantages of natural solvents.

## Figures and Tables

**Table 1:** Physical properties of natural oils used in the study.

	Sweet Almond Oil	Grapeseed Oil	Flaxseed Oil
density *	0.91 g/cm^3^	0.96 g/cm^3^	0.93 g/cm^3^
viscosity **	58 cP	44 cP	30 cP

* experimentally determined according to ISO 18301:2014 [31]. ** experimentally determined by HAAKE Viscotester rotational viscometer.

**Table 2:** Extraction equilibrium of 7-ACA (pH = 4.5) and TOA dissolved in dichloromethane at 25 °C.

TOA Concentration, g/L	TOA Concentration, mol/L	$ln\left[R_{3}N\right]$, mol/L	$ln\left(\frac{K_{D}}{\alpha_{A^{-}}}\right)$	Extraction Efficiency, %	$K_{D}$	$K_{E}$
10	0.0282	−3.565751	0.354079	43.715	0.776	15.18 × 10^4^ L/mol
20	0.0565	−2.872604	1.643681	73.825	2.820	
40	0.1131	−2.179456	2.352137	85.136	5.727	
80	0.2262	−1.486309	2.422979	86.010	6.148	
120	0.3393	−1.080844	2.441266	86.229	6.261	

**Table 3:** Extraction equilibrium of 7-ACA (pH = 4.5) and TOA dissolved in grapeseed oil at 25 °C.

TOA Concentration, g/L	TOA Concentration, mol/L	$ln\left[R_{3}N\right]$, mol/L	$ln\left(\frac{K_{D}}{\alpha_{A^{-}}}\right)$	Extraction Efficiency, %	$K_{D}$	$K_{E}$
10	0.0282	−3.565751	0.053172	36.502	0.574	0.61 × 10^4^ L/mol
20	0.0565	−2.872604	0.595852	49.726	0.989	
40	0.1131	−2.179456	1.055702	61.038	1.566	
80	0.2262	−1.486309	1.153325	63.333	1.727	
120	0.3393	−1.080844	1.158034	63.442	1.735	

**Table 4:** Extraction equilibrium of 7-ACA (pH = 4.5) and TOA dissolved in sweet almond oil at 25 °C.

TOA Concentration, g/L	TOA Concentration, mol/L	$ln\left[R_{3}N\right]$, mol/L	$ln\left(\frac{K_{D}}{\alpha_{A^{-}}}\right)$	Extraction Efficiency, %	$K_{D}$	$K_{E}$
10	0.0282	−3.565751	−0.301108	28.743	0.403	0.15 × 10^4^ L/mol
20	0.0565	−2.872604	0.269185	41.639	0.713	
40	0.1131	−2.179456	0.554310	48.688	0.948	
80	0.2262	−1.486309	0.648317	51.038	1.042	
120	0.3393	−1.080844	0.652691	51.147	1.046	

**Table 5:** Extraction equilibrium of 7-ACA (pH = 4.5) and TOA dissolved in flaxseed oil at 25 °C.

TOA Concentration, g/L	TOA Concentration, mol/L	$ln\left[R_{3}N\right]$, mol/L	$ln\left(\frac{K_{D}}{\alpha_{A^{-}}}\right)$	Extraction Efficiency, %	$K_{D}$	$K_{E}$
10	0.0282	−3.565751	−0.867195	18.633	0.229	0.03 × 10^4^ L/mol
20	0.0565	−2.872604	−0.555508	23.825	0.312	
40	0.1131	−2.179456	−0.258798	29.617	0.420	
80	0.2262	−1.486309	−0.128468	32.404	0.479	
120	0.3393	−1.080844	−0.091287	33.224	0.497	

## Data Availability

Data are contained within the article and Supplementary Materials.

## Supplementary Materials

The following supporting information can be downloaded at: https://www.mdpi.com/article/10.3390/biom15101371/s1, Figure S1: 7-ACA calibration curve.
