# Supplementary figures and images for: Natural Oils as Green Solvents for Reactive Extraction of 7-Aminocephalosporanic Acid: A Sustainable Approach to Bioproduct Recovery in Environmental Biotechnology
Source: Biomolecules. 2025 Sep 26;15(10):1371. doi: 10.3390/biom15101371 (PMC12563663; doi:10.3390/biom15101371)

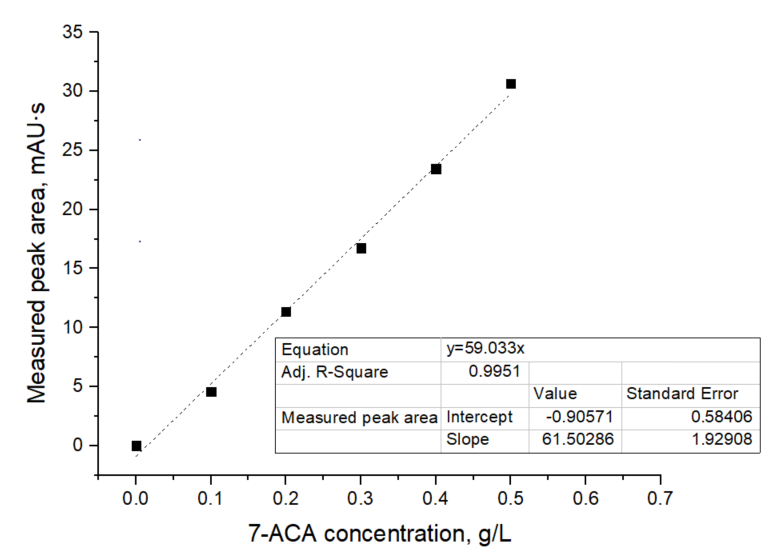

Supplement: Supplementary file 1 [file biomolecules-15-01371-s001.zip › biomolecules-3872048-supplementary.png]
